# The Impact of the COVID-19 Pandemic on Medical Imaging Case Volumes in Aseer Region: A Retrospective Study

**DOI:** 10.3390/medicines8110070

**Published:** 2021-11-12

**Authors:** Magbool Alelyani, Ali Alghamdi, Nasser Shubayr, Yazeed Alashban, Hajar Almater, Sultan Alamri, Ahmad Joman Alghamdi

**Affiliations:** 1 Department Radiological Sciences, King Khalid University, Abha 61421, Saudi Arabia; maalalyani@kku.edu.sa (M.A.); hajaralmater21@gmail.com (H.A.); 2 Department of Radiological Sciences, The University of Tabuk, Tabuk 47713, Saudi Arabia; alighamdi02@gmail.com; 3 Diagnostic Radiology Department, College of Applied Medical Sciences, Jazan University, Jazan 45142, Saudi Arabia; nshubayr@jazanu.edu.sa; 4 Medical Research Center, Jazan University, Jazan 45142, Saudi Arabia; 5 Department of Radiological Sciences, College of Applied Medical Sciences, King Saud University, Riyadh 11362, Saudi Arabia; Yalashban@ksu.edu.sa; 6 Department of Radiological Sciences, College of Applied Medical Sciences, Taif University, Taif 21944, Saudi Arabia; a.joman@tu.edu.sa

**Keywords:** COVID-19, medical imaging, radiology, Saudi Arabia

## Abstract

COVID-19 has had a significant impact on global health systems. The aim of this study was to evaluate how imaging volumes and imaging types in radiology departments have been affected by the COVID-19 pandemic across different locations. Methods: Imaging volumes in the Aseer region (in the south of Saudi Arabia) across main hospitals were reviewed retrospectively including all cases referred from different locations (outpatient, inpatient and emergency departments). Data for years 2019 and 2020 were compared. The mean monthly cases were compared using a *t*-test. Results: The total imaging volumes in 2019 were 205,805 compared to 159,107 in 2020 with a 22.7% overall reduction. A substantial decline was observed in both the April to June and the July to September periods of approximately 42.9% and 44.4%, respectively. With respect to location, between April and June, the greatest decline was observed in outpatient departments (76% decline), followed by emergency departments (25% decline), and the least impact was observed in inpatient departments, with only 6.8% decline over the same period. According to modality type, the greatest decreases were reported in nuclear medicine, ultrasound, MRI, and mammography, by 100%, 76%, 74%, and 66%, respectively. Our results show a statistically significant (*p*-value ≤ 0.05) decrease of cases in 2020 compared to 2019, except for mammography procedures. Conclusion: There has been a significant decline in radiology volumes due to COVID-19. The overall reduction in radiology volumes was dependent on the stage/period of lockdown, location, and imaging modality.

## 1. Introduction

Coronavirus disease (COVID-19) is a viral respiratory disease that was detected In December 2019. Its spread across the world since was first revealed in Wuhan, China [1]. In March 2020, the World Health Organization announced that COVID-19 was a global pandemic and must be tackled. The first case in the Kingdom of Saudi Arabia was recorded on 2 March 2020, as announced by the Ministry of Health. Governments have begun implementing many changes that affect daily life to limit the spread of the virus, such as social distancing, but it has been challenging in the Kingdom of Saudi Arabia (KSA), due to the level of civilization, its social and religious norms, and its annual hosting of religious mass gatherings [2]. These changes have ranged from closing schools and public facilities and announcing quarantine periods [3]. On the healthcare side, hospitals and other health care centers around the world have been forced to postpone elective procedures, including surgery. In addition, non-urgent procedures have been re-scheduled to ensure that resources are available for COVID-19 patients [4].

Due to the basic diagnostic function of CT and X-ray scans, radiology departments have an invaluable role in the management of COVID-19 patients [5]. Radiology departments have provided specific instructions for the effective use of imaging in emergency, inpatient, and outpatient locations. These instructions have been introduced mainly to reduce the risk of infection transmission and contamination of equipment during the scanning of COVID-19 patients [4]. These precautions and instructions have had a significant impact on the imaging volumes and finances of medical imaging departments [6]. In one study, conducted in New York City, the researchers compared medical imaging cases from many different hospitals, from 1 January 2019, pre-COVID-19, to 18 April 2020, post-COVID-19. This study showed a total reduction of 28% in the number of imaging cases performed [4]. In other research conducted in California, it was noted that volumes of imaging decreased in emergency departments after the announcement of the quarantine period in comparison with baseline daily mean volumes in 2019 [7].

Some studies have been performed specifically on one type of medical imaging modality. For example, a retrospective review of consecutive CT head examinations ordered through the ED during the early stages of the COVID-19 pandemic in Canada (12 March–8 April 2020) was performed and compared with examinations undertaken during the pre-COVID-19 period (12 February–10 March 2020). The average daily volume of CT head examination orders decreased significantly during COVID-19 compared with pre-COVID-19 [8]. For breast imaging, the COVID-19 pandemic was shown to have influenced the volume of mammography examinations in a retrospective study of retrieved records for patients receiving mammography services in southern Taiwan. The test period (22 weeks) of the COVID-19 pandemic was between the 1st and 22nd week in 2020. The pre-COVID-19 pandemic control period was between the 1st and 22nd week of 2019 [9].

The aim of our study was to assess the impact of the COVID-19 pandemic on the total number of imaging cases performed in the Aseer region. In addition, we sought to assess the extent of increase or decrease in the volume of cases and to ascertain which types of imaging modality were most affected by the COVID-19 pandemic. To our knowledge, there is no published study describing the impact of the pandemic on radiology departments in Saudi Arabia. This study may contribute to the preparation of plans for a gradual return and continuation of services of radiology departments during the crisis.

## 2. Materials and Methods

Study design: we performed a retrospective review of imaging case volumes in the main hospitals in the Aseer region (Aseer Central Hospital, Military Hospital, and Khamis Mushait General Hospital). The study period for 2020 was selected to encompass the peak in COVID-19 cases and to capture the subsequent impacts on imaging case volumes, including all cases in 2020 compared with 2019. 

Data collection and extraction: In cooperation with the supervisors of the picture archiving and communication system (PACS) system in the selected hospitals, imaging case numbers were provided for each month for 2019 and 2020. The imaging cases were further classified according to department (i.e., emergency department, inpatients, and outpatients), and modality types (e.g., X-ray, ultrasound, computed tomography [CT], interventional radiology, magnetic resonance imaging [MRI], nuclear medicine, and mammography). 

Statistical analysis: Statistical assessments were performed using the Statistical Package for the Social Sciences (version 20; SPSS-Inc.; New York, NY, USA) considering 95% confidence intervals (α = 0.05); *t*-tests and one-way ANOVA were applied to the data. 

## 3. Results

**Figure 1:**
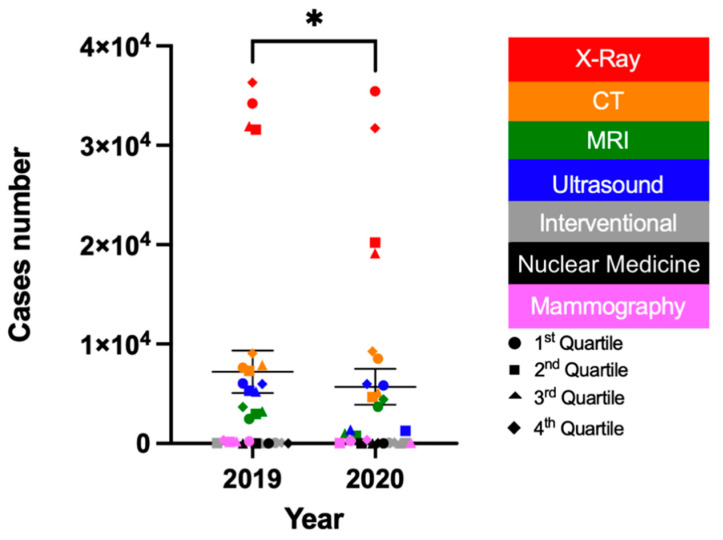
All performed cases at radiology departments using different modalities in 2019 and 2020. The asterisk (*) denotes a significant reduction between the number of performed cases in 2020 compared to 2019.

**Figure 2:**
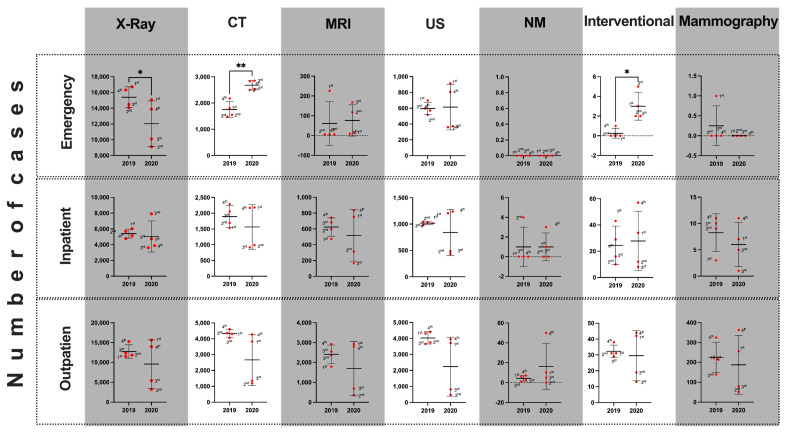
A comparison of the total number of cases performed by different imaging modalities (columns) at different departments (rows) between 2019 and 2020. CT= Computed Tomography, MRI= Magnetic Resonance Imaging, US= Ultrasound, NM= Nuclear Medicine.

**Figure 3:**
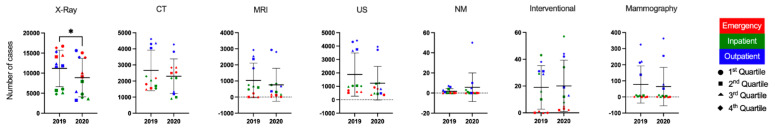
The total number of cases divided according to quartiles.

The total number of imaging scans performed at all medical centers in 2020 from January to December was 318,2014. This was in comparison to 404,170 scans in 2019 for the equivalent time period (Figure 1 and Table 1). Further analysis showed a statistically significant reduction of 21.27% (*p* = 0.02) in 2020 compared with 2019. In addition, the number of emergency(ER) cases revealed a significant reduction in the X-ray department in 2020 compared to 2019 (Figure 2 and Figure 3). Of interest is that the ER cases in the CT and interventional departments showed a significant increase in 2020.

Table 1 and Table 2 represent the imaging cases by patient service locations and imaging modality types in the years 2019 and 2020. Closer inspection of the tables shows a reduction in the total number of cases in the second and third quartiles of 2020 compared with 2019. This reduction was significant, approaching 40%, as shown in Table 2. Finally, Table 3 shows the comparison of the 2019 and 2020 mean monthly medical imaging cases for the imaging modality types by patient service location. The results show a statistically significant (*p*-value ≤ 0.05) decrease of cases in 2020 compared to 2019, with the exception of mammography procedures.

## 4. Discussion

This study investigated the impact of COVID-19 on total radiological volumes for different locations and modalities in the Aseer (southern) region in Saudi Arabia. On 23 March, the Ministry of Health in Saudi Arabia announced a total lock down across the country to limit the spread of COVID-19 among the Saudi population. Since then, there has been a dramatic overall decline of radiology practices, as reported in our study. 

The results from our study showed an overall 21.27% reduction of imaging volumes in 2020 compared to 2019 for all radiological practices. The greatest decline was observed in the April–June and July–September periods, with approximately 42.9% and 44.4% decline, respectively. This reported decline was consistent with anecdotal observations that suggested that imaging practices could anticipate a 50% to 70% decrease in radiological volumes, lasting a minimum of 3 to 4 months, depending on the severity of the COVID-19 pandemic in each region [1]. Naidich, Jason J., et al. investigated the impact of the COVID-19 pandemic on the number of cases performed. They reported an overall reduction of 28% in the total imaging volume including all types of imaging modalities in all service locations [4].

The extent of decline in imaging volumes in our study varied in different locations. A decline in radiology volumes was expected during the COVID-19 pandemic, especially for outpatients [4]. In our study, the greatest decline was observed in outpatient departments (for April–June) by 76%, followed by emergency departments at 25%, with the least impact on imaging observed for inpatient cases, with an increase in 2020 by 6.8%. A previous study obtained similar findings with outpatient departments experiencing the greatest declines of about 88%, followed by emergency departments (46% decline), with the least impact on inpatient department imaging volumes (4% decline) (2). In another study, ED imaging volumes reduced by 32 to 40% during a two week time period after the lockdown compared to those of 2019 [7]. 

This remarkable decrease in the imaging volumes in ED may be explained by so-called “superusers”, with the finding that 12% of ED patients consume 50% of all ED imaging services annually [10]. Christey et al. reported a considerable decrease (43%) in the total volume of all injury admissions during level-4 lockdown during the COVID-19 pandemic [11]. Lazzerini et al., reported a reduction in pediatric cases, where parents avoided accessing hospitals due to fear of infection [12]. The correlation between fear of infection and access to health care systems was reported previously during the SARS pandemic in 2003 [13]. 

One explanation offered for the considerable reduction in ED imaging cases is that as a result of adherence to the instructions of the Ministry of Health to stay at home during the lockdown to limit the spread of COVID-19, the risk of traffic and workplace accidents was much reduced. However, an observational study by Agarwal and his colleagues (2020) about the impact of the COVID-19 pandemic on the use of ER CT head contradicts this [8]. Despite the overall decrease of examinations performed, the result of CT head imaging for acute cases increased. This was consistent with another study in which no change in stroke admissions was observed during the early weeks of pandemic in ED [14]. 

Despite the short period since the start of COVID-19, the impact of the pandemic on health care organizations has been reported globally. For example, a previous French study showed a remarkable decrease in acute stroke cases (34%), and seizures (36%) in ED, while another USA study found a 39% decline in stroke imaging records [15,16]. Modality type also influenced the rate of decline, with the greatest decreases reported in nuclear medicine, ultrasound, MRI, and mammography by 100%, 76%, 74% and 66%, respectively. Similarily, Naidich, Jason J., et al. found that the greatest decrease was reported in mammography (94%), followed by nuclear medicine (85%), and then MRI (74%) [4]. 

Chou, Chen-Pin, et al. retrospectively studied the impact of the COVID-19 pandemic on mammography volumes in Taiwan [9]. Compared to 2019, they found a total decline of 37% of mammographs (3041 vs. 4816, *p* < 0.001). Mammography volume decline also varied by imaging type. From pre-COVID-19 to COVID-19, self-requested, screening, and diagnostic mammography examinations declined by 96%, 51%, and 6% respectively. These results indicated that women weighed the advantages of mammography imaging against the risk of infection, reflecting awareness that they should avoid delaying diagnosis which may worsen cancer outcomes, especially for symptomatic women.

Our results suggest that there is a correlation between imaging volumes and human behaviour following the lockdown, and variation in the number and findings of imaging examinations performed in this period. We believe that our study can provide valuable information to assist health service providers in understanding how a pandemic can impact epidemiology and the type and volumes of radiological examinations performed during this period.

## 5. Conclusions

We conclude that the announcement of complete lockdown in Saudi Arabia due to the COVID-19 pandemic has led to significant decline in radiology volumes. The overall reduction in radiology services was dependent on the stage/period of lockdown, location, and imaging modality, with greater influence in the first period (April–June), outpatient departments, and nuclear medicine, respectively. In future work, this study could be extended to assess post-COVID-19 changes.

## Figures and Tables

**Table 1:** Imaging cases by patient service location and imaging modality type in years 2019 and 2020. CT= Computed Tomography and MRI= Magnetic Resonance Imaging.

Year	2019				2020			
Months	January–March	April–June	July–September	October–December	January–March	April–June	July–September	October–December
Total volume	50,681	47,337	48,599	55,468	53,860	27,042	27,043	51,162
Patient location								
Emergency	19,431	16,128	16,564	19,062	18,533	12,075	13,486	17,528
Inpatient	9119	9051	8875	8844	8936	9669	5188	8190
Outpatient	22,131	22,158	23,160	27,562	26,391	5298	8326	25,444
Modality type								
X-ray	34,213	31,570	31,941	36,323	35,434	20,213	19,123	31,718
CT	7641	7296	7903	9073	8546	4676	5118	9276
MRI	2484	2965	3236	3672	3694	788	1022	3646
Ultrasound	6046	5313	5240	5989	5834	1275	1669	5992
Interventional	74	41	45	68	79	33	26	103
Nuclear Medicine	6	5	3	7	11	0	5	53
Mammography	217	147	231	336	262	57	80	374

**Table 2:** Percent difference in medical imaging cases between 2020 and 2019 for the same period according to patient service location and imaging modality type.

Year	Percent Change in 2020 Imaging Volumes Compared to 2019			
Months	January–March	April–June	July–September	October–December
Total volume	+6.27%	−42.87%	−44.35%	−7.76%
Patient location				
Emergency	−4.62%	−25.13%	−18.58%	−8.05%
Inpatient	−2.01%	+6.83%	−41.54%	−7.39%
Outpatient	+19.25%	−76.09%	−64.05%	−7.68%
Modality type				
X-ray	+3.57%	−35.97%	−40.13%	−12.68%
CT	+11.84%	−35.91%	−35.24%	+2.24%
MRI	+48.71%	−73.42%	−68.42%	−0.71%
Ultrasound	−3.51%	−76%	−68.15%	+0.05%
Interventional	+6.76%	−19.51%	−42.22%	+51.47%
Nuclear Medicine	+83.33%	−100%	+66.67%	+657.14%
Mammography	+20.74%	−61.22%	−65.37%	+11.31%

**Table 3:** Comparison of the 2019 and 2020 mean monthly medical imaging cases for each imaging modality type by patient service location.

Year	2019		2020		*p*-Value
	Mean	95% CI	Mean	95% CI	* ≤ 0.05
Total volume	7217	2820–11,614	5701	2014–9389	0.02
Emergency Department					
X-ray	15,386	13,293–17,479	12,033	7441–16,625	0.02 *
CT	1753	1257–2249	2680	2381–2978	0.01 *
MRI	61	−114–236	76.5	−48.89–201.9	0.86
Ultrasound	595.5	484.2–716.8	613.5	153.3–1074	0.89
Nuclear Medicine	0	0–0	0	0–0	N/A
Interventional	0.25	−0.54–1.04	3	0.74–5.25	0.04 *
Mammography	0.25	−0.54–1.04	0	0–0	0.39
Inpatient					
X-ray	5402	4498–6305	5037	1892–8183	0.7
CT	1895	1348–2441	1565	438.4–2692	0.45
MRI	624	438.8–809.2	517.5	−1.92–1037	0.59
Ultrasound	1019	973.8–1063	841	141.8–1540	0.47
Nuclear Medicine	1	−2.18–4.18	1	−1.25–3.25	>0.99
Interventional	24.5	1.16–47.83	27.75	−8.21–63.72	0.73
Mammography	8.25	2.53–12.62	6	−0.62–12.62	0.47
Outpatient					
X-ray	12,724	9983–15,465	9552	−240.8–19,344	0.25
CT	4331	3965–4696	2659	88.71–5230	0.1
MRI	2404	1648–3160	1694	−472.1–3859	0.41
Ultrasound	4033	3426–4640	2238	−701–5177	0.09
Nuclear Medicine	4.25	−0.13–8.63	16.25	−20.14–52.64	0.33
Interventional	32.25	25.97–38.53	29.5	4.35–54.64	0.7
Mammography	224.3	101.9–346.6	187.3	−47.85–422.4	0.47
All Patient Service Locations					
X-ray	11,171	8272–14,069	8874	5839–11,909	0.04 *
CT	2659	1856–3463	2301	1619–2984	0.39
MRI	1030	346.4–1713	762.5	113.3–1412	0.31
Ultrasound	1882	858.4–2906	1231	435.1–2027	0.08
Interventional	19	8.64–29.36	20.08	7.89–32.27	0.75
Nuclear Medicine	1.75	0.09–3.4	5.75	−3.31–14.81	0.28
Mammography	77.58	4.14–151	64.42	−11.28–140.1	0.39
